# FAN1 Activity on Asymmetric Repair Intermediates Is Mediated by an Atypical Monomeric Virus-type Replication-Repair Nuclease Domain

**DOI:** 10.1016/j.celrep.2014.06.001

**Published:** 2014-06-26

**Authors:** Simon Pennell, Anne-Cécile Déclais, Jiejin Li, Lesley F. Haire, Wioletta Berg, José W. Saldanha, Ian A. Taylor, John Rouse, David M.J. Lilley, Stephen J. Smerdon

**Affiliations:** 1 Division of Molecular Structure, MRC National Institute for Medical Research, The Ridgeway, London NW7 1AA, UK; 2 CRUK Nucleic Acids Structure Research Group, College of Life Sciences, University of Dundee, Dundee DD1 5EH, UK; 3 Division of Mathematical Biology, MRC National Institute for Medical Research, London NW7 1AA, UK; 4 MRC Protein Phosphorylation Unit, College of Life Sciences, University of Dundee, Dundee DD1 5EH, UK

## Abstract

FAN1 is a structure-selective DNA repair nuclease with 5′ flap endonuclease activity, involved in the repair of interstrand DNA crosslinks. It is the only eukaryotic protein with a virus-type replication-repair nuclease (“VRR-Nuc”) “module” that commonly occurs as a standalone domain in many bacteria and viruses. Crystal structures of three representatives show that they structurally resemble Holliday junction resolvases (HJRs), are dimeric in solution, and are able to cleave symmetric four-way junctions. In contrast, FAN1 orthologs are monomeric and cleave 5′ flap structures in vitro, but not Holliday junctions. Modeling of the VRR-Nuc domain of FAN1 reveals that it has an insertion, which packs against the dimerization interface observed in the structures of the viral/bacterial VRR-Nuc proteins. We propose that these additional structural elements in FAN1 prevent dimerization and bias specificity toward flap structures.

## Highlights

• Bacterial proteins comprising solely a VRR-Nuc domain are dimeric in solution
• Bacterial VRR-Nuc domains act as Holliday-junction-resolving enzymes
• A conserved helical insertion in the FAN1 VRR-Nuc domain prevents dimerization
• FAN1 monomers cannot cleave Holliday junctions and instead cleave 5′ flap DNA

## Graphical Abstract

**Figure:**
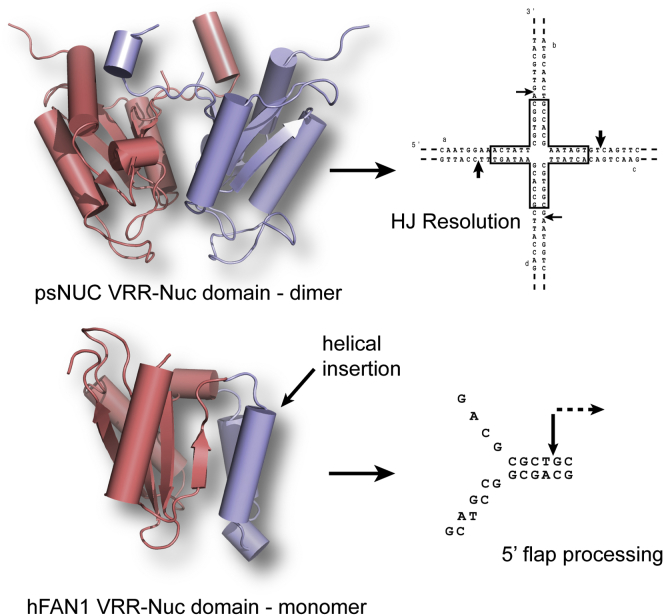

## Teaser

The Fanconi anemia pathway is responsible for clearing DNA interstrand crosslinks that block DNA replication and transcription leading to genome instability. Here, Pennell et al. characterize the Fanconi-anemia-associated nuclease FAN1, comparing the structure and activity of its catalytic VRR-Nuc domain with prokaryotic examples. FAN1 is monomeric with 5′ flap specificity, whereas prokaryotic VRR-Nuc domains are dimeric Holliday-junction-resolving enzymes. FAN1 is proposed to contain a conserved helical insertion blocking dimer formation and consequently restricting substrate specificity.

## Introduction

FANCD2/FANCI-associated nuclease 1 (FAN1, hereafter hFAN1) is a structure-specific nuclease required for the repair of interstrand DNA crosslinks (ICL) and is recruited to sites of DNA damage in a manner dependent on the monoubiquitinated form of FANCD2 (Kratz et al., 2010, Liu et al., 2010, MacKay et al., 2010, Smogorzewska et al., 2010). Cells lacking FAN1 display increased sensitivity only to agents that induce ICLs. Mutations in humans in some of the genes involved in ICL repair cause Fanconi anemia, a rare chromosome instability disorder (Kottemann and Smogorzewska, 2013). Mutations in FAN1 have not been identified in FA patients but have been associated with karyomegalic interstitial nephritis, a form of chronic kidney disease (Zhou et al., 2012). In addition, *FAN1* has been identified as a susceptibility gene for schizophrenia and autism (Ionita-Laza et al., 2014).

hFAN1 has previously been shown to preferentially cleave branched DNA structures that mimic intermediates of DNA repair, with a strong preference in vitro for 5′ flap DNA. hFAN1 also possesses a 5′-3′ exonuclease activity (Kratz et al., 2010, MacKay et al., 2010, Smogorzewska et al., 2010). All FAN1 nuclease activities are mediated by a virus-type replication-repair nuclease (“VRR-Nuc”) domain located at the C terminus. This domain, which is conserved in all FAN1 orthologs, is a member of the ancient restriction endonuclease-like superfamily, with a predicted four-stranded mixed α/β fold with αβββαβ topology (Iyer et al., 2006). The active site of the members of this superfamily of enzymes has a conserved PDX_n_(D/E)XK motif, which in most cases is able to coordinate divalent metal ions required for activity (Steczkiewicz et al., 2012). VRR-Nuc domains were first identified through a combination of sequence homology and secondary structure prediction (Kinch et al., 2005). FAN1 is the only VRR-Nuc-domain-containing protein in eukaryotes, although there are many examples in bacteria and bacteriophage (MacKay et al., 2010), and in most of these cases, VRR-Nuc domains are present as single-domain proteins. Here, using a combination of structural and biochemical approaches allied to molecular modeling, we show how the oligomeric state of FAN1 orthologs and standalone VRR-Nuc domain proteins are directly related to their activities on asymmetric versus symmetric DNA substrates, respectively.

## Results

### Processing of 5′ Flaps Is Conserved among FAN1 Orthologs

**Figure 1:**
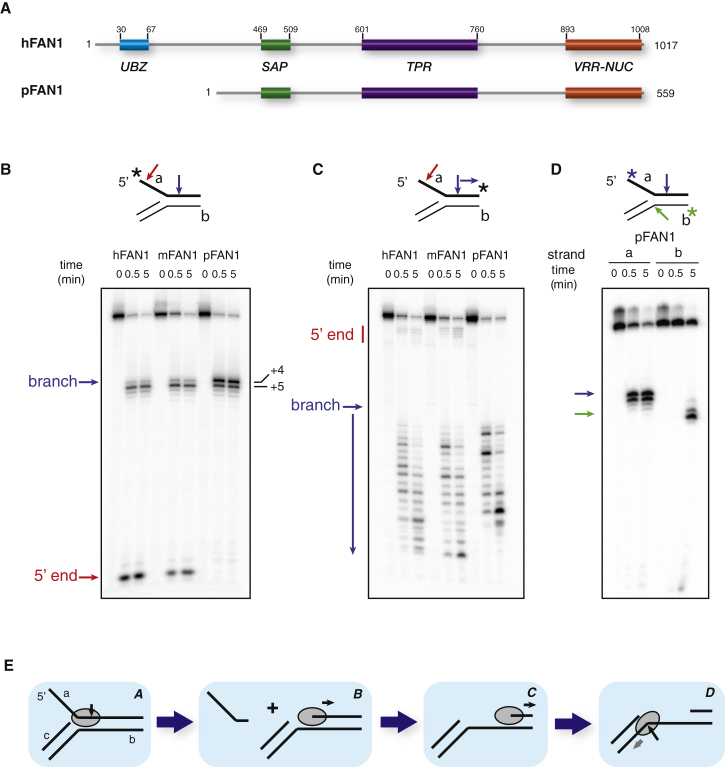
FAN1Domain Schematic and Catalytic Activity (A) Domain organization of hFAN1 and pFAN1. hFAN1 domain boundaries from Smogorzewska et al. (2010). (B) Endonucleolytic cleavage of 5′-labeled 5′ flap DNA by human FAN1 and orthologs. (C) 5′-3′ exonucleolytic cleavage of 3′-labeled 5′ flap DNA by human FAN1 and orthologs. (D) Progression of a- and b-strand 5′ flap cleavage by pFAN1. (E) Schematic of the proposed mechanism of pFAN1 activity on 5′ flaps.

Our initial aim was to crystallize and biochemically characterize FAN1, and to this end, we purified a range of FAN1 orthologs from human, mouse, and *Pseudomonas* (hFAN1, mFAN1, and pFAN1, respectively; Figure 1A), expressed in bacteria. To compare their nuclease activities, proteins were incubated with 5′ flap substrates labeled at either the 5′ (Figure 1B) or 3′ end (Figure 1C) of the flap strand. Figure 1B shows a similar cleavage pattern for hFAN1 and mFAN1, generating a long endonucleolytic product corresponding to cleavage 4 bp after the branchpoint and a short product (4 nt) corresponding to cleavage close to the 5′ end of the flap. This short product was the major band for hFAN1 but was less dominant for mFAN1. pFAN1 exhibited a different profile with strong cleavage 4 and 5 bp after the branchpoint but with no detectable cleavage at the 5′ end. However, all three enzymes displayed some 5′ to 3′ exonuclease activity following the initial cut (Figure 1C), suggesting that they differ in their ability to bind and/or cleave DNA ends, but not in their ability to process DNA branchpoints. As reported in MacKay et al. (2010), the FAN1 proteins do not have activity against Holliday junction (HJ) substrates (Figure S1A).

The apparent lack of pFAN1 activity at DNA ends suggested it as a good candidate for a more-detailed investigation of branchpoint processing. To this end, pFAN1 was incubated with 5′ flap substrates labeled at the 5′ end of either the flap strand (a) or the duplex strand (b) (Figures 1D and S1B). Whereas the a strand was cleaved almost to completion within the first minute, significant cleavage of the b strand only became apparent after 2 min, proceeding to completion in ∼40 min. The different kinetics of these two cleavage events suggests that the 5′ flap substrate is initially cleaved on the a strand and processed into a gapped duplex or a 5′ overhang, which is then targeted by another pFAN1 molecule in a slower reaction (Figure 1E).

### VRR-Nuc Domains Are Structurally Related to Holliday-Junction-Resolving Enzymes

**Figure 2:**
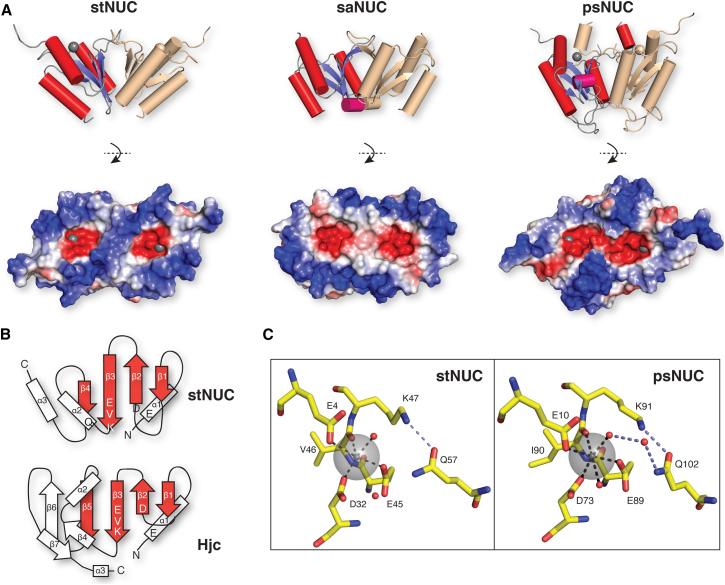
Crystal Structures of Prokaryotic VRR-Nuc Domain Proteins (A) Structures of stNUC, saNUC, and psNUC dimers. α helices in red, 3_10_ helices pink, and β sheets blue. In each case, the dimeric partner is shown in light pink. Adjacent are electrostatic representations of the active site surface with magnesium atoms as gray spheres if present. (B) Topology diagrams demonstrating similarity between the structures of stNUC and Hjc. Core secondary structural elements are in red and active site residues highlighted. (C) Active site of stNUC (left) and psNUC (right).

Despite considerable effort, we have thus far failed to crystallize FAN1 or isolate soluble VRR-Nuc domains from any of the FAN1 orthologs. However, we were able to solve the first VRR-Nuc domain structures using single-domain proteins derived from three bacteria and bacteriophage as surrogates: *Psychrobacter* (psNUC); *Streptococcus equi* bacteriophage P9 (stNUC); and *Salmonella enteritidis* typing phage 3 (saNUC) (Figure 2A). Crystallographic statistics are presented in Figure S2A.

stNUC was solved by single-wavelength anomalous diffraction (SAD) methods on crystals of selenomethione-substituted protein and refined against native 1.3 Å resolution data (Figures 2A and S2B). One molecule is present per asymmetric unit (AU) and comprises a central four-stranded β sheet surrounded by three α helices in a αβββαβα topology (Figure 2B). The protein crystallized as a dimer with monomers related by a crystallographic 2-fold axis and interacting through a substantial hydrophobic interface formed by the central β sheets of opposing molecules and bounded on either side by the α2 helix from each partner. Electrostatic surface representations highlight the position of the negatively charged active sites on each monomer (Figure 2A), which bind a single magnesium ion in a geometry that is distorted from a classical octahedral arrangement toward a square pyramidal configuration (Figure 2C). K47 is presumably involved in positioning the catalytic water molecule as would be expected from homology with other members of the restriction endonuclease-like superfamily (Knizewski et al., 2007). Q57, a conserved residue within VRR-Nuc domains (http://pfam.xfam.org/family/vrr_nuc), makes a hydrogen bond to K47 that is absent in archaeal Holliday junction resolvase (HJR) structures. The active sites are surrounded by positively charged residues in a figure-of-eight pattern, as seen in *Sulfolobus solfataricus* Hjc (Protein Data Bank [PDB] ID 1HH1; Bond et al., 2001), consistent with a role in substrate DNA recognition and orientation.

saNUC was solved by molecular replacement using the stNUC structure as a search model (Figure 2A), revealing a dimeric arrangement of two molecules in the AU essentially identical to that seen in the stNUC crystals. Despite only having 34% sequence identity with stNUC, both have core structures that are remarkably similar (root-mean-square deviation = 1.3 Å over 86 Cα) and the orientation of active site residues is essentially identical to stNUC.

psNUC was solved by SAD and refined against 2.0 Å resolution data. Here, the crystals contain six molecules per AU, comprising three dimers that, like those of saNUC and stNUC, form around a central hydrophobic β sheet interface. There are, nonetheless, several elaborations (Figure 2A). In particular, the linker between β1-β2 forms a partially structured extension over the active site surface. Such structures are common in Holliday-junction-resolving enzymes, including RecU (PDB ID 1ZP7), and likely play a role in the binding and orientation of substrate (Cañas et al., 2011). Hexacoordination of a magnesium ion is seen at each active site, involving the E-PD-EXK motif and two water molecules in a configuration stabilized by the conserved Q102 (Figure 2C). The active site surface is bounded by a ring of positively charged residues to allow substrate recognition (Figure 2A).

DALI searches (Holm and Sander, 1995) show that the structures with greatest similarity to the VRR-Nuc domains are all Holliday-junction-resolving enzymes exemplified by Hjc (Figure 2B). This observation raised the possibility that dimeric VRR-Nuc domain proteins selectively process four-way DNA junctions, and we therefore investigated the nuclease activities of these three proteins.

### VRR-Nuc Domains Are Dimeric and Cleave Four-Way DNA Junctions

**Figure 3:**
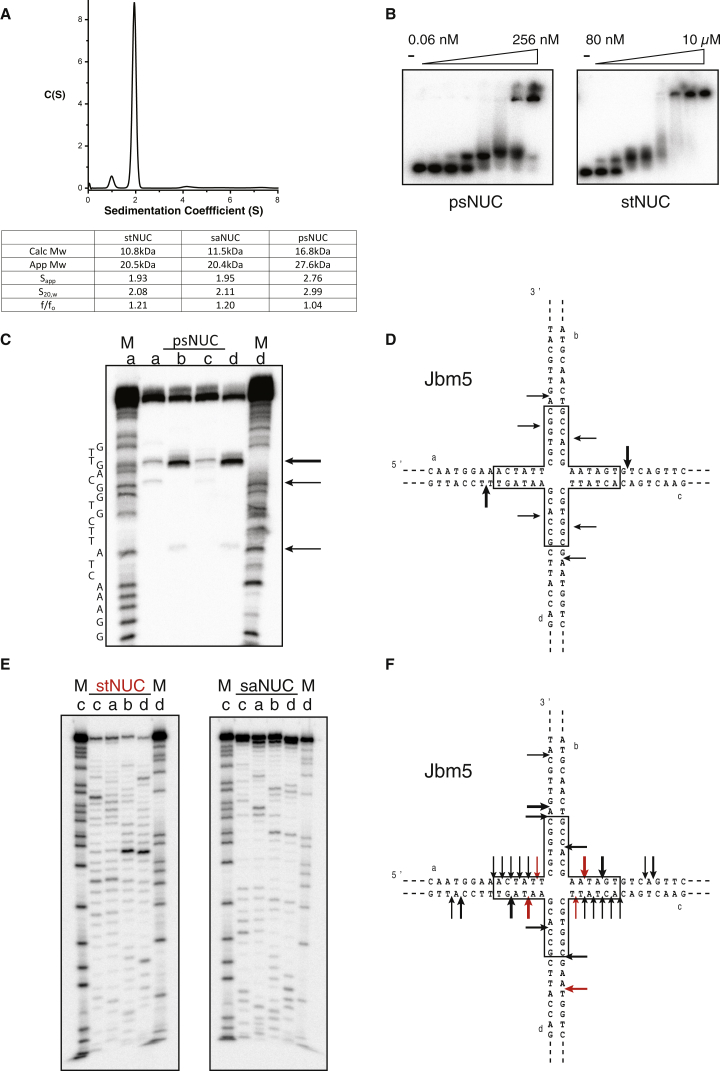
Characterization of Prokaryotic VRR-Nuc Domain Proteins (A) AUC sedimentation velocity of stNUC demonstrating a dimeric species with table of VRR-Nuc AUC-derived parameters inset. (B) Gel mobility shift assay of psNUC and stNUC with HJ substrates. (C) Cleavage of a HJ substrate by psNUC. (D) psNUC cleavage sites mapped onto the HJ secondary structure. (E) Cleavage of a HJ substrate by stNUC and saNUC. (F) stNUC (red arrows) and saNUC (black arrows) cleavage sites mapped onto the HJ secondary structure.

A key characteristic of canonical HJRs is that they bind and process their substrate as dimers to achieve substrate recognition and productive resolution and predominantly form homodimers in solution in the absence of substrate (Lilley and White, 2001). We examined the quaternary structure of the VRR-Nuc domains in solution by performing analytical ultracentrifugation (AUC) experiments (Figure 3A). For each protein, the main peak in the sedimentation coefficient distribution analysis (C(S)) corresponded to a dimer. For psNUC, an additional minor peak corresponding to a tetrameric species was present at concentrations above 70 μM. This agrees well with our crystal structures and the known quaternary structure of HJRs.

The ability of VRR-Nuc domains to bind a range of DNA structures was examined using electrophoretic mobility-shift assays. As shown in Figure 3B, psNUC bound a four-way DNA junction stoichiometrically with an affinity indicative of a subnanomolar equilibrium dissociation constant (*K*_D_) typical of HJRs (∼1 nM; White et al., 1997) whereas stNUC bound the junction over 100 times more weakly (*K*_D_ = 200 nM). Nevertheless, in both cases, the binding was structure-selective, with psNUC forming weaker complexes with a 5′ flap and a nicked three-way junction and stNUC binding the four-way junction only (Figure S3A). At high protein concentrations, higher-order complexes formed with equal efficiency on all substrates. By contrast to the other proteins, saNUC only formed nonspecific, higher-order complexes.

We next investigated the catalytic activity of the VRR-Nuc domains toward these DNA substrates. Figure 3C shows the cleavage products obtained with psNUC and Jbm5, a four-way junction that can undergo 12 steps of branch migration such that each possible dinucleotide can be accommodated at the branchpoint (Kvaratskhelia et al., 2001). This junction was cleaved symmetrically, and we observed only two discrete cleavage sites on each strand that occurred within or close to the homologous core of the junction (boxed, Figure 3D). This resulted in the formation of ligatable nicked duplex DNA, diagnostic of productive HJ resolution (Figure S3B). Under the same assay conditions, we observed no cleavage of linear double-stranded DNA substrate and very weak activity on most branched DNA structures with the exception of replication forks, which were cleaved to an intermediate degree (Figure S3C). Finally, psNUC cleaved a fixed HJ 3 nt 3′ to the branchpoint, and this activity was inhibited by the presence of a slow-cutting mutant of T7 endonuclease I, suggesting that they compete for the branchpoint (Figure S3D).

stNUC and saNUC exhibited weaker nuclease activity against the Jbm5 HJ substrate, detectable only at protein concentrations above 1 μM and with increased reaction times (Figure 3E). Under these conditions, stNUC showed robust activity, whereas cleavage by saNUC was much reduced. This suggests that our saNUC preparations have a low active fraction potentially also explaining the lack of structure selectivity seen in the gel mobility shift experiments. In both cases, we observed a range of symmetrical cleavage sites within or close to the homologous core of the junction as well as some nonsymmetrical sites away from the branchpoint (Figure 3F). stNUC also selectively cleaved a nicked three-way junction (RF) and a 3′ flap structure (3′F) 2 bp 3′ to the branchpoint (Figure S3E). Both enzymes additionally exhibited a nonspecific nuclease activity producing a ladder of cleavage products (Figure S3F).

psNUC therefore not only structurally resembles an archaeal HJR but also behaves as one, displaying binding to and symmetrical cutting of HJ substrates with affinities and cleavage patterns typical of a canonical HJC protein. Whether the levels of activity observed for stNUC and saNUC are representative of those in vivo is unclear, but it would appear unlikely given their close structural homology to psNUC. Crucially, however, both enzymes retain the ability to produce symmetric cleavages within a HJ substrate, an activity that is not characteristic of VRR-Nuc domains within the FAN1 orthologs.

### FAN1 Enzymes Are Monomeric in Solution

The isolated VRR-Nuc domains studied here form dimers, consistent with an important role in the recognition of four-way DNA junctions, whereas the FAN1 orthologs exhibited no activity on four-way DNA junctions, instead processing 5′ flaps very efficiently. This suggested that the FAN1 VRR-Nuc domains may act as monomeric enzymes, and this hypothesis was investigated further.

**Figure 4:**
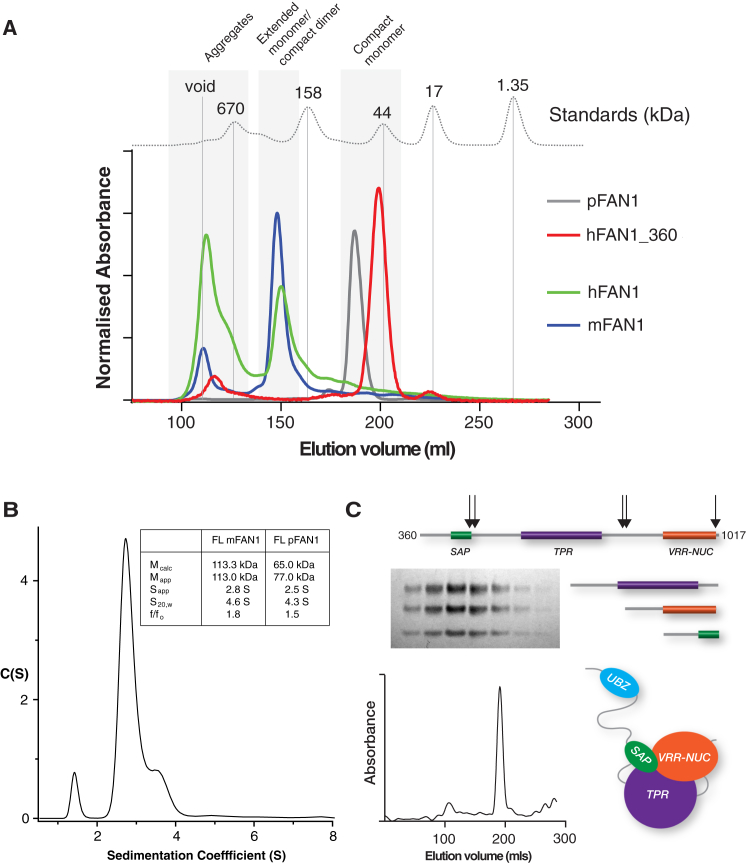
FAN1 Oligomeric Status (A) Overlay of S200 gel filtration traces of FAN1 orthologs against molecular weight standards. (B) AUC sedimentation velocity run of mFAN1 at 4°C demonstrating a monomeric species with table of FAN1 AUC-derived parameters inset. (C) S200 gel filtration trace of trypsin-treated hFAN1_360 showing the elution of a single protein peak. An SDS-PAGE gel of fractions across the peak is inset demonstrating the presence of three coeluting protein fragments.

Using analytical gel filtration, column retention times of hFAN1 and mFAN1 were consistent with a molecular weight of approximately 200 kDa, raising the possibility of a dimeric interaction (Figure 4A). By contrast, the column retention time of pFAN1 was consistent with a monomer. PSIPRED secondary structure predictions (Jones, 1999) suggest that both hFAN1 and mFAN1 are essentially unstructured over the first ∼370 residues, potentially explaining the low retention times in gel filtration experiments. To test this, we removed the first 359 residues of hFAN1 leaving the region of homology to pFAN1. This protein (hFAN1_360) was fully functional in nuclease assays (not shown) and eluted from gel filtration with a retention time consistent with a monomer. Although it remained possible that the N-terminal regions within mouse and human FAN1 might be mediating self-association, sedimentation velocity analysis showed unambiguously that, like pFAN1, full-length mouse FAN1 is monomeric (Figure 4B). Finally, limited tryptic proteolysis of hFAN1_360 produced three protected fragments identified by mass spectrometry as amino acids 360–507, 515–790, and 795–1,012. Gel filtration performed on a preparative scale tryptic digest gave rise to a single eluted peak containing three species with a retention time almost identical to the undigested molecule (Figure 4C). Indeed, attempts to express constructs corresponding to the tryptic fragments either in isolation or as pairs were unsuccessful, suggesting an intimate association of all three domains. Together, these results support the hypothesis that eukaryotic FAN1 proteins comprise an unstructured N terminus and a compact C terminus comprised of three domains that broadly correspond to the SAP, TPR, and VRR-Nuc homology regions.

### Modeling of FAN1 VRR-Nuc Domains Explains Their Oligomeric State and Substrate Specificity

**Figure 5:**
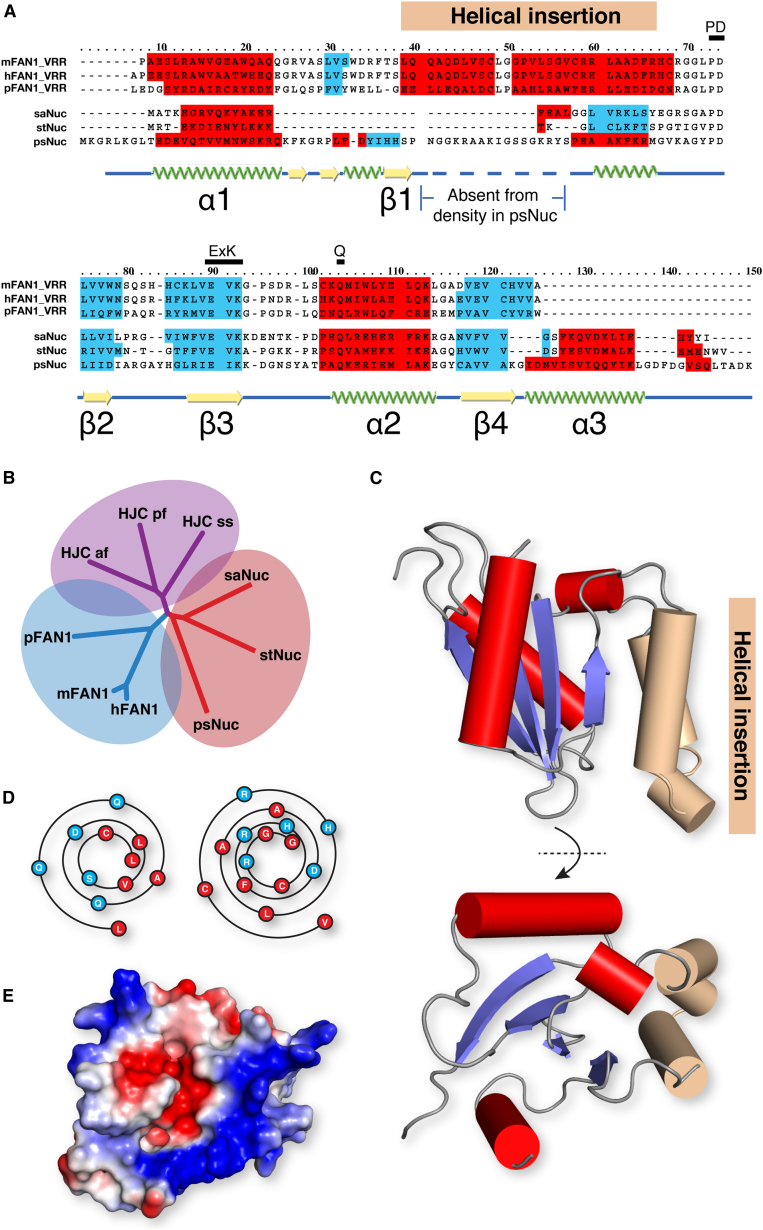
VRR-Nuc Domain Phylogeny and Modelling (A) PRALINE alignment of VRR-Nuc structures against FAN1 homologs overlaid with PSIPRED secondary structure predictions with α helices in red and β sheets in blue. The secondary structure of psNUC derived from the crystal structure is shown beneath. The position of the modeled helical insertion is shown above in light pink. Conserved catalytic residues are highlighted above. (B) Phylogenetic tree showing VRR-Nuc domains, FAN1 orthologs, and archaeal HJC sequences. (C) In silico modeling of the hFAN1 VRR-Nuc domain from the side (top) and top (bottom) colored according to the VRR-Nuc domain crystal structures. The predicted helical insertion is in light pink. (D) Helical wheel Wenxian diagrams of helix 1 (left) and helix 2 (right) of the hFAN1 helical insertion clearly showing hydrophobic and hydrophilic faces of each helix. (E) Electrostatic surface representation of the hFAN1 VRR-Nuc domain model in the same orientation as the bottom image in (C) showing the negatively charged active site (red) and positively charged DNA-binding surface (blue).

The apparent inability of the VRR-Nuc domain of FAN1 to dimerize could in principle result from its interactions with other regions of the full-length protein or be an intrinsic property of the domain. To further investigate this and reconcile the apparent difference in substrate specificity and oligomeric state between bacterially derived VRR-Nuc domains and the FAN1 proteins, we examined sequence alignments of VRR-Nuc domains from each group prepared using the PRALINE multiple alignment server (Simossis and Heringa, 2005; Figures 5A and S4A). The majority of bacterially derived VRR-Nuc domains conform to the αβββαβα topology seen in the crystal structure of stNUC. A subset of these proteins additionally contain a predicted short α-helical section of 8–11 residues between β1-β2, in an equivalent position to the α-helical section of the stalk extension of psNUC. For the FAN1 proteins, however, this is replaced by two strongly predicted α helices approximately 10 and 18 residues long separated by a conserved I/L-G/P dipeptide, giving rise to an αβααββαβ topology that is likely unique among the restriction endonuclease-like superfamily (Knizewski et al., 2007). This difference is also apparent from phylogenetic analysis of VRR-Nuc sequences where FAN1-derived VRR-Nuc domains populate a clade distinct from the bacterial and phage examples (Figures 5B and S4B).

To understand how this two-helix insertion might affect the quaternary structure of the FAN1 VRR-Nuc domain, we generated models of the VRR-Nuc domains from hFAN1 and pFAN1, both of which resemble the bacterial VRR-Nuc domains with respect to the β sheet and position of the active site residues (Figures 5C and S4C). Helical wheel representations of the helical insertion suggest that the insertion forms an amphipathic helix-turn-helix motif that packs onto the hydrophobic surface of the central β sheet, effectively blocking the canonical VRR-Nuc domain dimerization interface (Figure 5D). In keeping with the VRR-Nuc domain structures described in this study, the negatively charged active site pocket is surrounded by a ring of positive charge, which also extends along the outer surface of the helical insertion in our models (Figure 5E).

## Discussion

From the evidence presented above, it is clear that, at least in bacteria and bacteriophage, VRR-Nuc domains resemble Holliday-junction-resolving enzymes both structurally and functionally. The role of these domains in bacteriophage is unknown, but analogy with systems such as λ Rep, T7 endonuclease I, or T4 endonuclease VII suggests that they may be involved in DNA recombination, replication, and/or packaging (Sharples et al., 2004, White et al., 1997). As dimers, bacterial VRR-Nuc domains have two active sites and a DNA-binding surface with 2-fold symmetry that are well suited to the recognition and symmetrical, bilateral cleavage of Holliday junctions (Déclais and Lilley, 2008). In contrast, we now show that FAN1 proteins are monomeric, consistent with a different mode of asymmetric substrate selectivity. From our models, the presence of a highly conserved helical insertion between β1 and β2 suggests two direct effects on FAN1 VRR-Nuc domain function. First, the insertion occludes dimer formation by packing against the hydrophobic face of the central core β sheet. Second, the insertion alters the shape of the DNA-binding surface and provides a significant contribution to its overall positive charge, thereby influencing substrate preference. It is highly probable that these data explain why FAN1 cannot cleave Holliday junctions and instead prefers simple branched DNA species such as 5′ flaps.

Functional redundancy is a characteristic of DNA repair pathways with multiple structure-specific enzymes sharing common substrates and/or activities that are controlled by regulatory factors. For example, FAN1 and FEN1 share both 5′ flap endonuclease and 5′-3′ exonuclease activity (Harrington and Lieber, 1994). The relationship between FAN1 and the bacterial examples of VRR-Nuc domain described above bears some comparison to the relationship of FEN1 and GEN1, another member of the Rad2/XPG family. FEN1 and GEN1 each comprise a bipartite nuclease domain and a DNA-binding helix-turn-helix domain. FEN1 preferentially targets 5′ flap structures as a monomeric enzyme. GEN1 is a monomer in solution and cleaves 5′ flaps and replication forks as a monomer but can form a dimer on Holliday junctions to ensure their productive resolution by symmetrical, bilateral cleavage (Rass et al., 2010). In this case, GEN1 possesses gain-of-function modifications to the FEN1 architecture to allow dimerization in the presence of an appropriate substrate (Tsutakawa et al., 2011). Conversely, the FAN1 VRR-Nuc domain has evolved a loss-of-function modification to prevent dimerization. Nonetheless, it is tempting to speculate that reorganization of the helical insertion, either by binding to an accessory protein or posttranslational modification, could trigger formation of FAN1 dimers. In this scenario, the hydrophobic faces of the helical insertions could pack together to form a stalk structure over the active site surface as seen in RecU, where it plays a role in HJ orientation (Cañas et al., 2011).

Although FAN1 is required for ICL repair, it is not clear what step is controlled by FAN1. ICL repair involves homologous recombination (Kee and D’Andrea, 2010), and several nucleases have been identified that are thought to be responsible for resolution of the resulting Holliday junctions: SLX1 and MUS81 tethered to the SLX4 scaffold, together, and GEN1 (Castor et al., 2013, Wyatt et al., 2013). From this point of view, there is no need to invoke another resolvase activity. However, we cannot exclude the possibility that, under certain circumstances, FAN1 could be modified in a way that would enable dimerization and HJ cleavage by this nuclease in vivo.

## Experimental Procedures

### Protein Preparation

Clones of psNUC, stNUC, and saNUC (Uniprot accession numbers: A5WF35, A7J283, and A3EZR7, respectively) were synthesized by GeneART and subcloned into pGEX 6P-1. Human, mouse, and *Pseudomonas aeruginosa* FAN1 clones in pGEX 6P-1 were prepared by the DNA-cloning service at the University of Dundee. Proteins were expressed as glutathione S-transferase fusions in *E. coli* and purified by means of glutathione-Sepharose affinity chromatography. Proteins were cleaved off the affinity column with 3C protease and applied to Superdex 75 or 200 gel filtration in 300 mM NaCl, 20 mM Tris (pH 8), and 0.5 mM tris(2-carboxyethyl)phosphine (TCEP), depending on the target protein size. FAN1 protein purification required an additional heparin step to remove DNA contamination prior to gel filtration.

### Crystallography

Crystals were obtained by the sitting drop vapor diffusion method using the Morpheus crystallization screen (Molecular Dimensions; Supplemental Information). Diffraction data were collected at 100 K on beamline IO4 at the Diamond Light Source and processed with Denzo/Scalepack (HKL Research). The structures of selenomethionine containing derivatives of stNUC and psNUC were determined by automated SAD/model building using PHENIX (Adams et al., 2010). saNUC was solved by molecular replacement with PHENIX using stNUC as a search model. Models were improved and refined using Coot, Refmac, and PHENIX.

### Activity Assays

DNA substrates were prepared as in MacKay et al. (2010) with the exception of Jbm5, prepared as in Kvaratskhelia et al. (2001). Purified recombinant proteins were preincubated with the radiolabelled substrate in binding buffer (Supplemental Information) at the reaction temperature for 10 min to allow binding to occur. The reaction was started by the addition of divalent metal ions and stopped by mixing with a 2-fold excess of EDTA. After addition of 66% formamide, the samples were heat denatured and analyzed by denaturing PAGE (12% polyacrylamide and 8 M urea in Tris-borate-EDTA [TBE] buffer). Gels were dried, exposed to storage phosphor screens, quantified by a Typhoon FLA 9500 (GE Healthcare) phosphorimager, and analyzed with the ImageQuant software.

### Gel Mobility Shift Assays

Varying amounts of purified recombinant protein were incubated with 1 nM radiolabelled substrate at 20°C for 30 min in binding buffer (Supplemental Information). Following addition of 2.5% Ficoll-400, samples were run on 8% native polyacrylamide gels in TBE buffer. Gels were dried, exposed to storage phosphor screens, quantified by a Typhoon FLA 9500 (GE Healthcare) phosphorimager, and analyzed with the ImageQuant software.

### Sedimentation-Velocity AUC

Sedimentation-velocity experiments were performed in a Beckman Optima Xli analytical ultracentrifuge, using aluminum double-sector sapphire cells in an An-50 Ti rotor. The rotor speed was 50,000 rpm, and the temperature was maintained at 277 K. Prior to centrifugation, protein samples were dialyzed exhaustively against the buffer blank (50 mM Tris/HCl [pH 8.0], 300 mM NaCl, and 0.50 mM TCEP). Interference images were collected every 180 s during the sedimentation run. The data recorded from moving boundaries were analyzed by the program SEDFIT in terms of both discrete species and continuous distribution function of sedimentation coefficient (*c*(*s*)) and molar mass (c(M)), allowing determinations of sedimentation coefficient and shape-independent molecular weight (Brown and Schuck, 2006).

### Model Building

The FAN1 VRR-Nuc domains (human, mouse, and *Pseudomonas*) have the same predicted secondary structure using PSIPRED (McGuffin et al., 2000) and therefore likely the same topology as standalone domains except for an insertion consisting of two confidently predicted helices. Twenty models of hFAN1 and pFAN1 were initially built based on the combined crystal structures of the archaeal HJR, HJC (PDB code 1GEF; Nishino et al., 2001), and the SdaI restriction endonuclease (PDB code 2IXS; Tamulaitiene et al., 2006) using the “modeler” option (Sali and Blundell, 1993) in QUANTA (Accelrys). The different models were calculated by varying the initial model and optimizing the objective function using conjugate gradients and molecular dynamics with simulated annealing using the CHARMm force field. The models with the lowest objective function were chosen for further refinement. This consisted of introducing the two helices insertion using the manual modeling options in QUANTA, taking care to ensure proper packing of hydrophobic residues.

## Author Contributions

S.P. and J.L. solved the structures of the VRR-Nuc domains and performed biophysical characterization. S.P. prepared the FAN1 proteins and performed biophysical characterization. A.-C.D. carried out all nuclease biochemical assays. W.B. assisted with protein preparation and crystallization. L.F.H. performed protein crystallization. J.W.S. performed computer modelling. I.A.T. assisted with biophysical characterization of all proteins. S.P., A.-C.D., J.R., D.M.J.L., and S.J.S. wrote the manuscript.

## Accession Numbers

Coordinates of the stNUC, saNUC, and psNUC structures along with associated structure factor amplitudes have been deposited with the Protein Data Bank with identifiers 4QBO, 4QBN, and 4QBL, respectively.

## Supplemental Information

Document S1. Supplemental Experimental Procedures and Figures S1–S4

Document S2. Article plus Supplemental Information
