# Supplementary material for: FAN1 Activity on Asymmetric Repair Intermediates Is Mediated by an Atypical Monomeric Virus-type Replication-Repair Nuclease Domain
Source: Cell Rep. 2014 Jun 26;8(1):84–93. doi: 10.1016/j.celrep.2014.06.001 (PMC4103454; doi:10.1016/j.celrep.2014.06.001)

Supplemental information for:

# FAN1 activity on asymmetric repair intermediates is mediated by an atypical monomeric VRR-Nuc domain

Simon Pennell[1‡*], Anne-Cécile Déclais[2‡], Jiejin Li[1‡], Lesley F. Haire[1], Wioletta Berg[1], José W. Saldanha[3], Ian A. Taylor[1], John Rouse[4], David M. J. Lilley[2], Stephen J. Smerdon[1]

[1] Division of Molecular Structure, MRC National Institute for Medical Research, The Ridgeway, London, NW7 1AA, UK
[2] CRUK Nucleic Acids Structure Research Group, College of Life Sciences, University of Dundee, Dundee, DD1 5EH, UK
[3] Division of Mathematical Biology, MRC National Institute for Medical Research, London, NW7 1AA, UK
[4] MRC Protein Phosphorylation Unit, College of Life Sciences, University of Dundee, Dundee, DD1 5EH, UK

**‡ These authors contributed equally to this work**

*** To whom correspondence should be addressed: (spennel@nimr.mrc.ac.uk)**

## Experimental Procedures

### Crystallisation conditions

For crystallisation, all proteins were concentrated in a buffer containing 300mM NaCl, 50mM Tris-HCl pH 7.0, 0.5mM TCEP and mixed 1:1 with well buffer in sitting drops at 18° C as below.

stNUC - 0.2M sodium citrate, 20% w/v PEG3350

stNUC-Se - 0.2M sodium citrate, 20% w/v PEG3350

saNUC - 2.2M ammonium sulphate, 0.1M Tris-HCl pH 8.0

psNUC-Se - 12.5 % w/v PEG 1000, 12.5% w/v PEG 3350, 12.5% v/v MPD, 0.02 M sodium L-glutamate, 0.02 M DL-alanine, 0.02 M glycine, 0.02 M DL-lysine, 0.2 M DL-serine, 0.1 M MES/imidazole pH 6.5

### Gel mobility shift assay and binding buffers

Optimised binding buffers were as follows:

FAN1 orthologues - 50 mM Tris-HCl (pH 8.0), 30 mM NaCl, 1 mM DTT, 0.1 mg/ml BSA; for psNUC - 20 mM Tris-HCl (pH 7.5), 50 mM NaCl, 1 mM DTT, 0.1 mg/ml BSA

stNUC - 20 mM Tris-HCl (pH 8.0), 50 mM NaCl, 1 mM DTT, 0.1 mg/ml BSA

saNUC - 20 mM Tris-HCl (pH 8.0), 100 mM NaCl, 1 mM DTT, 0.1 mg/ml BSA.

Binding assays were performed with 1 nM substrate and the protein concentration was varied from 62.5 pM to 256 nM in 4-fold increments for psNUC and from 78.1 nM to 10 $\mu$M in 2-fold increments for stNUC.

### Activity assay buffers and conditions

FAN1 orthologues (100 nM) were assayed with 10 nM substrate and 1 mM $MnCl_2$ at 30°C, psNUC (50 nM) with 5 nM substrate and 10 mM $MgCl_2$ at 25°C and stNUC and saNUC (5 $\mu$M) with 5 nM substrate and 10 mM $MgCl_2$ at 37°C.

### psNUC/T7 endonuclease I competition assay

Increasing amounts of the T7 endonuclease I D55A mutant were incubated with an h strand end labelled fixed HJ in binding buffer at 25°C for 5 min before adding 50 nM psNUC and assaying its activity as described in the main text.

### Ligation of psNUC reaction products

The ligation experiment was performed using a version of Jbm5 with one arm extended by 10 bp (Jbm-lig). Oligonucleotide sequences were as follows:

a: GCGTTACAATGGAAACTATTCGTGGCAGTTGCATCCAACG

b-lig: CGTTGGATGCAACTGCCACGAATAGTGTCAGTTCCAGACGCGGGGATCCG

c-lig: CGGATCCCCGCGTCTGGAACTGACACTATTCGTGGCGAATGGTCGTAAGC

d: GCTTACGACCATTCGCCACGAATAGTTTCCATTGTAACGC

This substrate was incubated with psNUC alone, T4 DNA ligase (New England Biolabs) alone or both enzymes simultaneously in 1X T4 DNA ligase buffer (New England Biolabs) supplemented with 50 mM NaCl. The reaction was stopped at various times with an excess of EDTA and the enzymes digested with proteinase K before analysing the products by denaturing PAGE.

# Supplementary Figures

## Figure Legends

**Figure S1 – Refers to Figure 1 - A.** Activity assay of pFAN1 against the Jbm5 HJ substrate labelled on the a (left) and b (right) strands demonstrating no activity **B.** Time course following pFAN1 5'-3' exonuclease activity on a 5' flap substrate labelled at the 3' end of the a (left) and b (right) strands.

**Figure S2 – Refers to Figure 2 - A.** Table of crystallographic statistics **B.** A portion of the 1.3Å resolution stNUC electron density (2Fo-Fc) map contoured at 2.0$\sigma$.

**Figure S3 – Refers to Figure 3 - A.** Gel mobility shift experiments demonstrating psNUC (left) and stNUC (right) structure-selective complex formation with Holliday junction substrates. (S – specific, NS – non-specific). **B.** Ligation experiment, showing that HJ cleavage by psNUC generates ligatable nicked DNA duplexes. Junction Jbm-lig was 5'-$^{32}$P-labelled on the 40 nt long d strand (thick line). Symmetrical cleavage by psNUC followed by nick ligation produces a radiolabelled 50-mer. The labelled substrate was incubated with no enzyme, psNUC, T4 DNA ligase or both enzymes for various times (indicated) and the reaction products analysed by denaturing PAGE. The lane on the far right is a 50-mer marker. A 50 nt product appears after incubation with both enzymes. **C**. psNUC displayed weak cleavage activity against a range of DNA substrates. Cleavage sites are indicated as arrows on the secondary structure diagrams below **D.** Competition experiment between psNUC and a slow-cutting mutant (D55A) of T7 endonuclease I (endo I), showing that psNUC targets the branchpoint of a four-way junction. A sample corresponding to a 2:1 endo I:psNUC ratio was also run alongside a chemical sequencing ladder in order to map the cleavage sites of both enzymes. The positions of cleavage sites are marked by blue (endo I) and black (psNUC) arrows on the diagram to the right. **E.** stNUC cleavage activity against a range of substrates. In order to distinguish structure specific cleavage events from non-specific nuclease activity (see panel F), this assay was performed in the presence of unlabelled calf-thymus DNA (0.1mg/ml) to reduce the background band intensity. Incubations were at 30˚C for 0, 15 or 60 minutes. **F.** stNUC cleavage of replication fork and Jbm5 HJ substrates demonstrating non-specific cleavage activity.

**Figure S4 – Refers to Figure 5 - A.** PRALINE sequence alignment of FAN1 and bacterial/bacteriophage VRR-Nuc sequences. **B.** N-J phylogenetic tree generated using Phylip based on PRALINE alignments of FAN1, viral/bacterial VRR-Nuc domains and HJC sequences. FAN1 proteins are in blue. HJCs are in green. VRR-Nuc domains are in orange with the subset of VRR-Nuc domains containing a short helical segment between β1 and β2 shown in red. **C.** In silico modelling of the VRR-Nuc domain from pFAN1 from the side (left) and top (right). α-helices are in red and β-sheets in blue. The predicted helical insertion is shown in light pink **D.** Electrostatic surface representation of the pFAN1 VRR-Nuc domain model from the top showing the active site surface (red region in the centre).

**Figure S1**

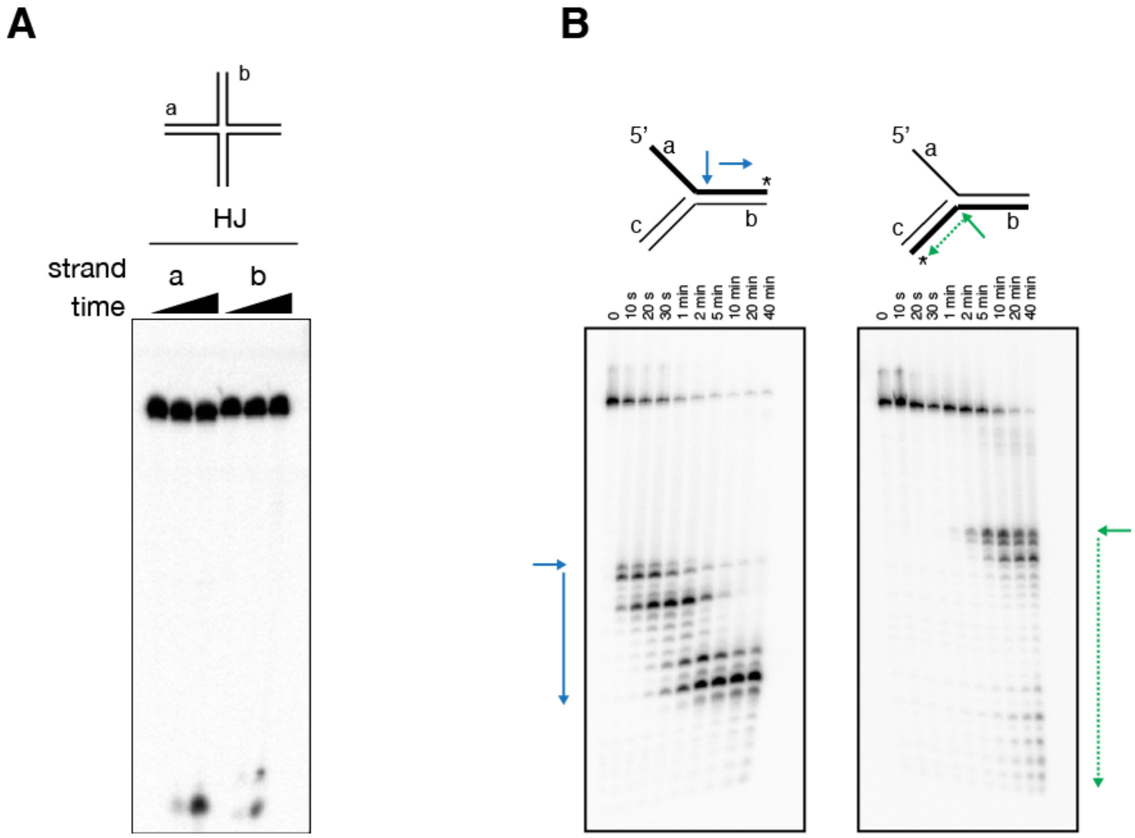
A
B
a
b
HJ
strand
a
b
time
5'
a
c
b
*
0
10 s
20 s
30 s
1 min
2 min
5 min
10 min
20 min
40 min

# Figure S2

## A

| | *stNUC -Se* | *stNUC* | *psNUC -Se* | *saNUC* |
|---|---|---|---|---|
| Protein Data Bank ID | | 4QBO | 4QBL | 4QBN |
| ***Data Collection*** | | | | |
| Resolution (Å) | 20.0 - 2.5 | 15.0 - 1.3 | 30.0 - 2.0 | 20.0 - 1.85 |
| Wavelength (Å) | 0.9791 | 0.9763 | 0.9804 | 0.9763 |
| Space group | $C222_1$ | $C222_1$ | C2 | F222 |
| a, b, c (Å) | 45.0, 60.0, 81.1 | 44.9, 60.5, 81.1 | 213.1, 51.2, 127.3 | 86.2, 99.3, 109.9 |
| α, β, γ (°) | 90.0, 90.0, 90.0 | 90.0, 90.0, 90.0 | 90.0, 125.5, 90.0 | 90.0, 90.0, 90.0 |
| Molecules per AU | 1 | 1 | 6 | 2 |
| No. reflexions: total | 74498 | 194996 | 733871 | 133292 |
| No. reflexions: unique | 7118 | 27396 | 147916 | 20335 |
| Completeness (%) | 97.0 (98.0) | 99.5 (99.3) | 100.0 (100.0) | 99.8 (99.4) |
| Redundancy | 10.5 (10.7) | 7.1 (6.8) | 5.0 (4.8) | 6.6 (6.1) |
| I/σI | 23.2 (26.1) | 19.8 (5.3) | 11.8 (2.0) | 28.7 (3.5) |
| $R_{merge}$ | 0.066 (0.085) | 0.074 (0.492) | 0.111 (0.686) | 0.061 (0.534) |
| ***Refinement*** | | | | |
| Resolution (Å) | | 15.0 - 1.3 | 30.0 - 2.00 | 20.0 - 1.85 |
| Reflections | | 27354 | 76117 | 20234 |
| $R_{work}$ | | 17.2 (33.1) | 17.5 (25.5) | 16.3 (19.9) |
| $R_{free}$ | | 18.0 (30.7) | 20.3 (31.1) | 19.7 (25.1) |
| rmsΔ bonds (Å) | | 0.011 | 0.008 | 0.018 |
| rmsΔ bond angles (°) | | 1.27 | 0.97 | 1.68 |
| ***Structure/Stereochemistry*** | | | | |
| No. atoms: non-H protein | | 2111 | 5821 | 1564 |
| No. atoms: Mg | | 1 | 6 | 0 |
| No. atoms: water | | 155 | 493 | 98 |
| Ramachandran: most favored | | 98.02 | 98.03 | 100 |
| Ramachandran: additionally allowed | | 1.98 | 1.55 | 0 |

## B

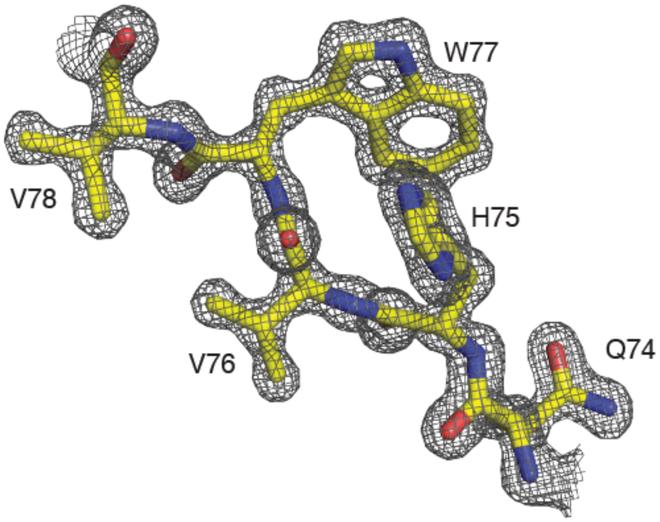

## Figure S3

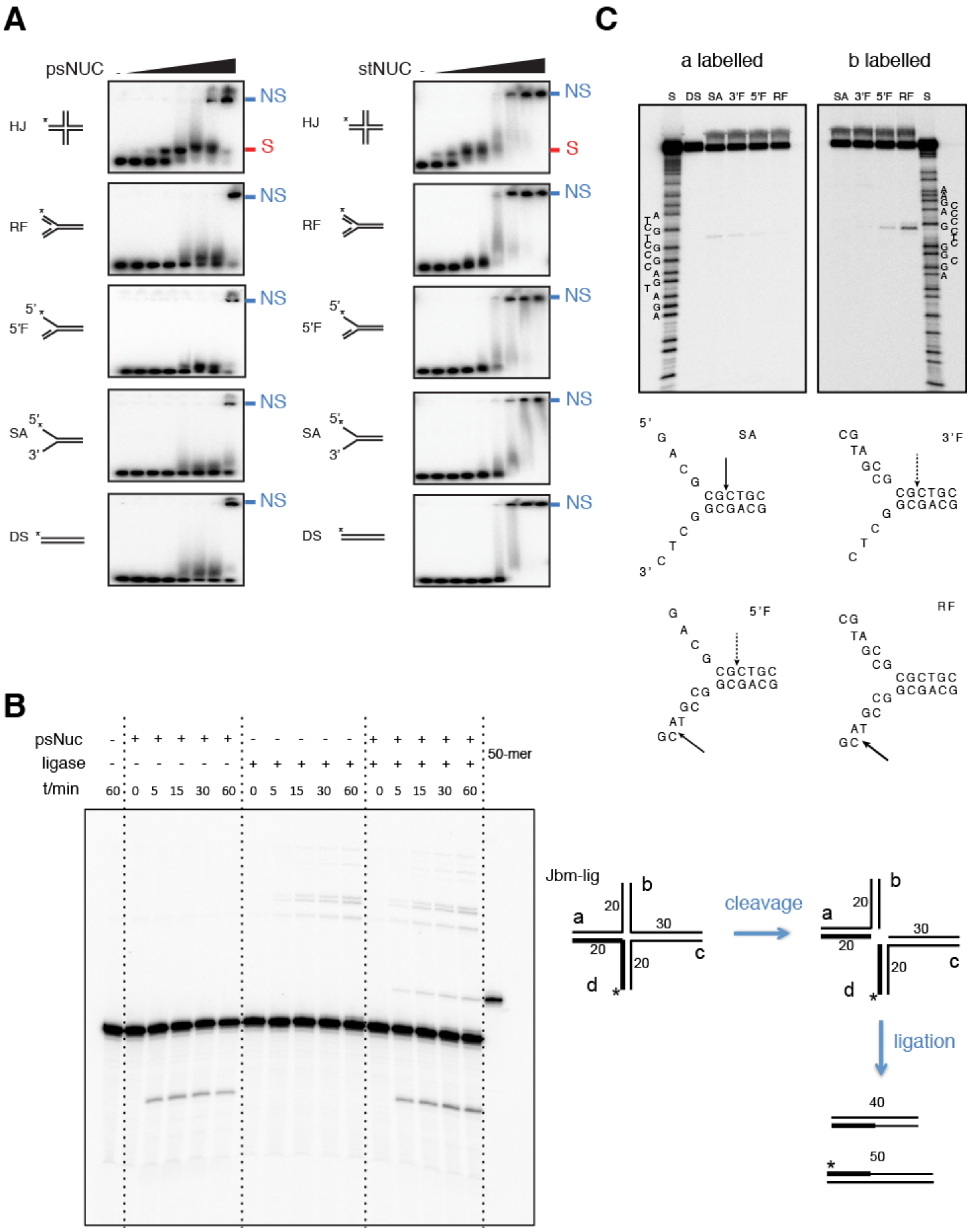
A
psNUC
stNUC
HJ
RF
5'F
SA
DS
NS
S
5'
3'
B
psNuc
ligase
t/min
60
0
5
15
30
60
50-mer
Jbm-lig
a
b
c
d
20
30
cleavage
ligation
40
50
C
a labelled
b labelled
S
DS
SA
3'F
5'F
RF

## Figure S3 (cont)

D

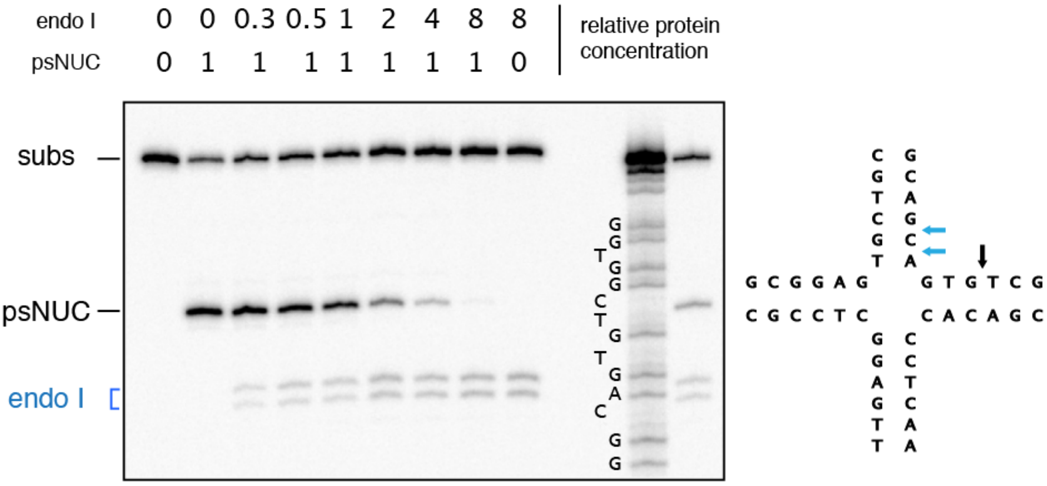


E

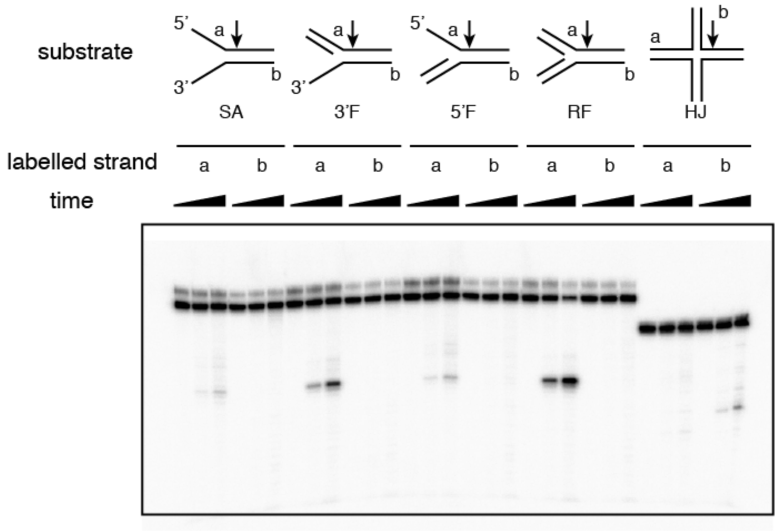


F

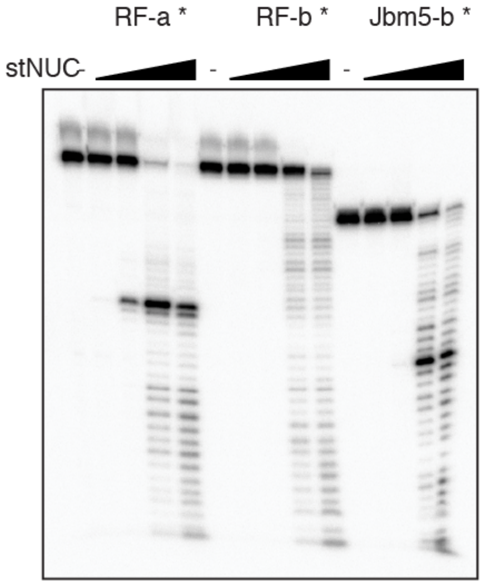

# Figure S4

## A

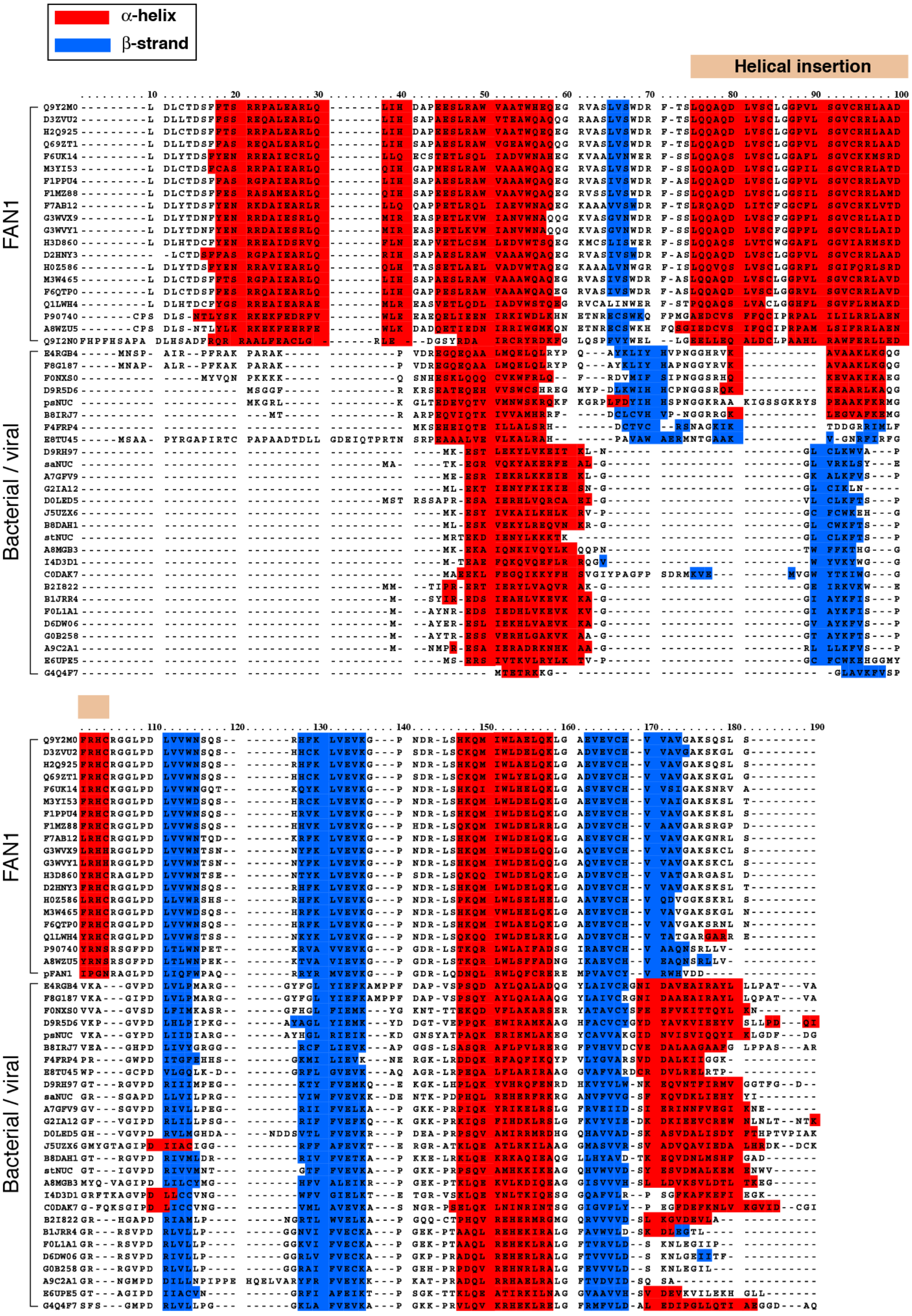

α-helix
β-strand
Helical insertion
FAN1
Bacterial / viral
FAN1
Bacterial / viral

## Figure S4 (cont)

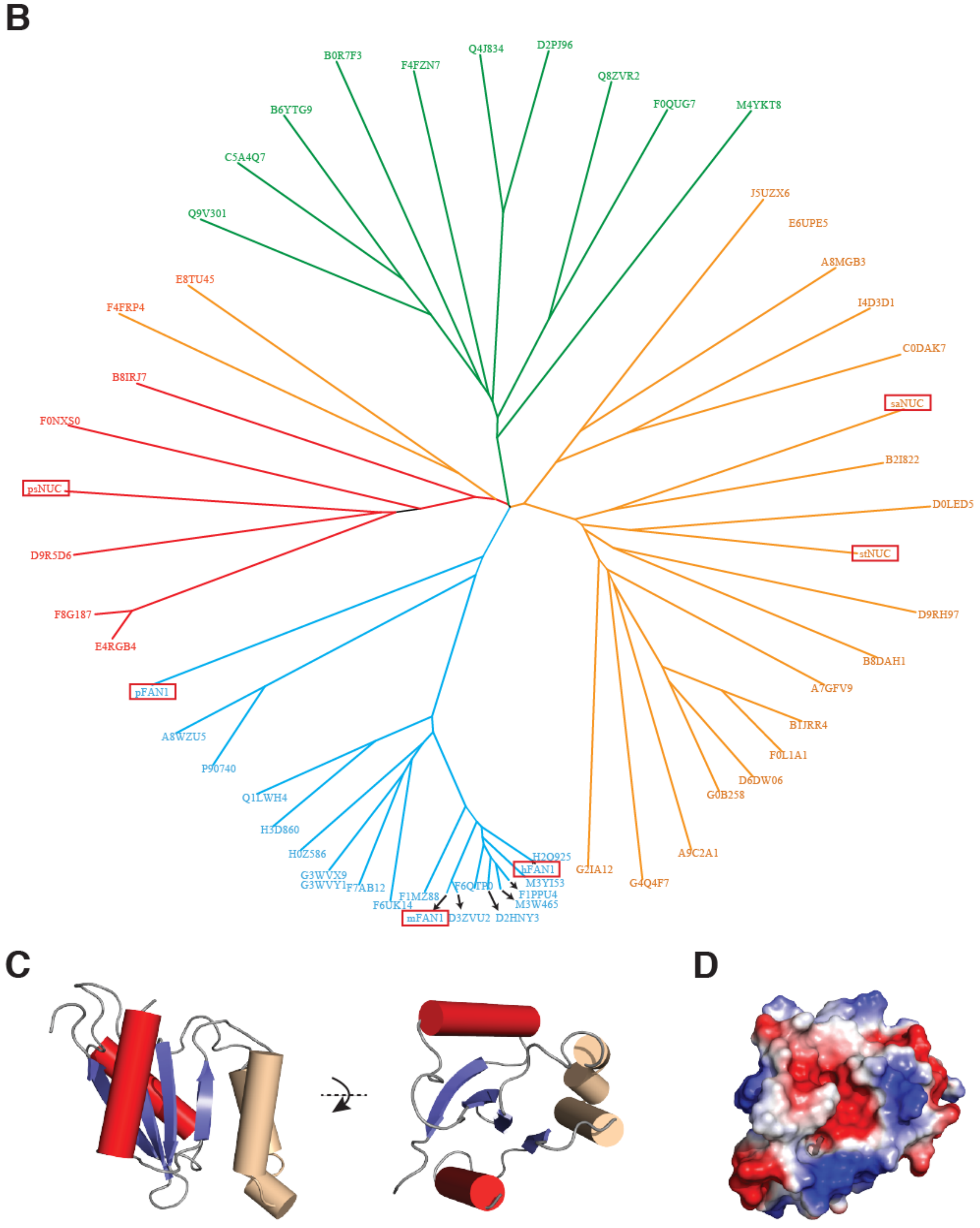

Supplement: Document S1. Supplemental Experimental Procedures and Figures S1–S4 [file mmc1.pdf]
